# The Clinical Value of Poor-Law Infirmaries

**Published:** 1915-01-23

**Authors:** 


### THE CLINICAL VALUE OF POOR-LAW INFIRMARIES.

### Their Lack of System and Suggested Reforms.

The laudable object of alleviating human suffering and curing disease is the *raison d'être* of every medical institution; but in addition certain other objects are aimed at, and one of the chief of these secondary objects is the desire of every properly administered institution to render itself of some clinical and scientific value and so justify its existence. That is the whole reason for recording in black and white clinical observations. It is done with an eye on the future. The records may prove useful to future statisticians, may elucidate later some obscure clinical point, or may prove of medico-sociological value in coming official publications (even after due allowance has been made for the personal factor).

To be of value, however, the recording, keeping, and classification of such records must be continued and systematic. One has only to pay a visit to most of the well-known voluntary hospitals in London to see the great care bestowed on such records carefully shelved, carefully numbered and indexed, and kept in a special room. Are the clinical records and notes of the cases in Metropolitan infirmaries as kept to-day of any value, either to the medical profession, to official departments, or to the general public? Such a question can be answered in the negative without fear of contradiction. Even where any notes are kept, they are of the scantiest description and practically valueless. Exceptionally one may find a carefully recorded clinical history and account of a case, but such finds are rarities. Even the treatment may be unrecorded, and the case may remain for months with only the original prescribed treatment recorded. There are no continued records, no deliberate system, no definite scheme, the few attempts are too spasmodic to be of any permanent value. Even in the most up to date of Metropolitan infirmaries such a state of affairs obtains.

Some time ago a junior medical officer of an infirmary was subpœnaed to a court case where damages were being claimed against a gas company for carbonic oxide poisoning from a defective fitting. This officer had never even seen the case and told the solicitors so; but they insisted on his presence, and he was required to produce the clinical records. In the witness-box he again repeated that he had never seen the case, but that the medical superintendent who was to be called for the opposing side had. The notes on production proved valueless. Their exact wording was as follows: "Admitted as a supposed case of gas poisoning; seems well enough now." No other observations implying that a thorough examination had been made appeared on the card, for this infirmary favours the bed-card system. The strange part of the proceeding was that such scanty records evoked no comment from judge or jury, not even from the counsel of the prosecutrix. Here was a case of some importance to a poor, perhaps destitute woman. She is nearly poisoned by gas, as medical evidence in the court proved; she was admitted to the infirmary to be treated for this, and her subsequent legal proceedings nearly failed because of the scanty infirmary records of the case, and the infirmary evidence was "on recollection." Had some strong and justly merited comments been made by one of the parties in court some improvement might have temporarily resulted; but the public are long-suffering.

Times, however, are changing. Panel doctors are being required by the Insurance Act to do a considerable amount of clerical work and to take notes and records of their diagnoses and treatment and number of visits—as data for possible future statistics. When the public awaken to the fact that such records are a necessity in any well-administered medical scheme, whether dealing with the destitute or not, the Poor-Law medical section will have to be prepared to answer the charge as to why such records have not been properly kept in the past.

Many autopsies are necessarily performed in such institutions; in nearly all the Metropolitan infirmaries even quite recently such a register as the *post-mortem* records did not exist. When it is remembered that many autopsies are performed on the Coroner's order, and the evidence thereat may involve the life of an accused person, it will be readily recognised how important it is that such autopsy observations should be permanently recorded in a book specially kept for the purpose, and signed by the medical man who performed the autopsy. Such a proceeding should have been insisted on long ago by the Local Government Board, and calls for the immediate attention of the Guardians of all Unions. There are only a few Poor-Law institutions in which annual reports are presented. The Metropolitan Asylums Board's medical institutions are an exception, and this fact proves the value of administration from one central source.

The defects of the absence of such clinical records are so apparent as to make comments needless. It helps to explain, however, part of the contempt of voluntary hospitals for infirmary work, and to some extent justifies the prejudice of a few poor against the rate-aided institution. It is difficult to say where the fault lies; certainly it is not with the individual officers. It is rather the system which obtains. In apportioning blame for lack of clinical records such facts must be considered, in addition to the well-known shortage of medical staff, the understaffing of the institution—really a medical starvation and consequent crippling of the institution—and the naturally resulting constant change. The vicious circle is quite a marked feature of present Poor-Law administration, and everything points to a faulty system, not to individual inefficiency. But the clinical records in infirmaries ought to receive more attention than they do, and, as is here shown, for more than immediate reasons.

